# Novel Multiplex Droplet Digital PCR Assays to Monitor Minimal Residual Disease in Chronic Myeloid Leukemia Patients Showing Atypical *BCR-ABL1* Transcripts

**DOI:** 10.3390/jcm9051457

**Published:** 2020-05-13

**Authors:** Jessica Petiti, Marco Lo Iacono, Matteo Dragani, Lucrezia Pironi, Cristina Fantino, Maria Cristina Rapanotti, Fabrizio Quarantelli, Barbara Izzo, Mariadomenica Divona, Giovanna Rege-Cambrin, Giuseppe Saglio, Enrico Marco Gottardi, Daniela Cilloni, Carmen Fava

**Affiliations:** 1 Department of Clinical and Biological Sciences, University of Turin, 10043 Turin, Italy; marco.loiacono@unito.it (M.L.I.); matteo.dragani@gmail.com (M.D.); giuseppe.saglio@unito.it (G.S.); daniela.cilloni@unito.it (D.C.); carmen.fava@unito.it (C.F.); 2 Division of Internal Medicine and Hematology, San Luigi Gonzaga Hospital, Orbassano, 10043 Turin, Italy; lucrezia.pironi@edu.unito.it (L.P.); c.fantino@gmail.com (C.F.); giovanna.rege@libero.it (G.R.-C.); enricogottardi@libero.it (E.M.G.); 3 Department of Oncohematology, Polyclinic of Tor Vergata, 00133 Rome, Italy; cristinarapanotti@yahoo.it (M.C.R.); madivona@gmail.com (M.D.); 4 Ceinge Advanced Biotechnologies Center, 80131 Naples, Italy; quarantelli@ceinge.unina.it; 5 Department of Molecular Medicine and Biotechnology, University Federico II & Ceinge Advanced Biotechnologies Center, 80138 Naples, Italy; barbara.izzo@unina.it

**Keywords:** chronic myeloid leukemia, *BCR–ABL1*, atypical transcripts, MRD monitoring, treatment-free remission, digital PCR

## Abstract

*BCR-ABL1* fusion transcript is the minimal residual disease marker in chronic myeloid leukemia; 2% of patients show unusual breakpoints generating atypical transcripts, not quantifiable by standardized real-time PCR (RT–PCR). Response monitoring is performed by non-quantitative NESTED PCR, useless for evaluating patients’ molecular remission, excluding them from treatment-free-remission protocols. Droplet digital PCR (ddPCR) is highly sensitive technology, allowing an absolute quantification independent of standard curves. Based on this, we have developed assays able to evaluate the molecular response in atypical patients. We designed new ddPCR-based molecular assays able to quantify atypical *BCR-ABL1* transcripts, with a detection limit of 0.001%, validated in a cohort of 65 RNA from 11 patients. Fifty samples were identified congruently by ddPCR and NESTED PCR (40 positives and 10 negatives for atypical *BCR–ABL1* transcript), while 11 positive samples were detected only by ddPCR. Our results highlight ddPCR usefulness, primarily when the *BCR–ABL1/ABL1* level is less than 1.5% and NESTED PCR results are often inaccurate. Furthermore, we identified 3 patients who maintained a deep molecular response for at least one year, who could be considered good candidates for treatment-free remission approaches. Here, we describe a new promising molecular approach, highly sensitive, to monitor atypical *BCR–ABL1* patients, paving the foundation to include them in treatment-free remission protocols.

## 1. Introduction

Chronic myeloid leukemia (CML) is characterized by a BCR–ABL1 fusion protein, originating from the translocation between chromosome 9 and 22 (t(9;22)), which is also the molecular marker for the evaluation of the minimal residual disease (MRD) in CML [1]. The majority of CML patients carry a “canonical” t(9;22) translocation characterized by chromosomal breakpoints located on exon 13 or 14 of the *BCR* gene and on exon 2 of the *ABL1* gene (respectively, the e13a2 and e14a2 fusion transcripts) [2]. However, in a small proportion of CML patients (1–2%), breakpoints on chromosomes 9 and 22 are located in unusual regions, giving rise to atypical rare transcripts, such as e13a3, e14a3, and e19a2. Quantitative real-time PCR (RT–PCR) is the standardized method for molecular response evaluation, but no assays have been set to quantify these rare transcripts [3]. Currently, MRD monitoring for patients that carry atypical transcripts is performed almost exclusively by non-quantitative methods, such as qualitative NESTED PCR [3]. The use of qualitative methods hinders the identification of the achievement of a major molecular response (MMR), which represents a fundamental prognostic factor for predicting progression and deciding therapy. In an era in which the response to tyrosine kinase inhibitors (TKI) is defined in terms of MMR or deep molecular response (DMR), it is difficult to establish the degree of response of patients with atypical transcripts in the absence of quantitative methodologies. Furthermore, in the last few years, several clinical discontinuation trials have demonstrated that 40–60% of chronic phase CML patients, who have achieved a stable DMR, can stop TKI without relapsing, reducing the treatment side effects, increasing CML patients’ life quality, and altogether decreasing the cost of therapy [4]. Currently, patients showing atypical transcripts are routinely excluded from the possibility of a treatment-discontinuation approach for safety reasons because of the inability to quantify their molecular response [5]. Recently, seven patients with atypical transcripts who have successfully discontinued their treatment due to severe toxicities were observed in clinical practice [6], also suggesting the feasibility of a treatment-free remission approach in these subjects. These observations highlight the need to develop new technologies for monitoring the disease status in atypical cases.

Droplet digital PCR (ddPCR) has recently emerged as a possible alternative or complement to RT-PCR to monitor low levels of disease [7,8]. ddPCR is based on water–oil emulsion droplet technology and, implementing PCR data with Poisson statistics, allows us to quantify the number of target molecules in a sample. Furthermore, ddPCR allows the absolute quantification of target molecules without the use of standard curves, and this characteristic seems appealing for the monitoring of molecular response. In comparison to RT–PCR, ddPCR has a higher reproducibility and may require a shorter standardization process [9].

In the present study, we developed a method for the evaluation of molecular response in CML patients characterized by atypical breakpoints based on ddPCR in order to improve the prognostic information that could allow TKI discontinuation and/or guide the therapeutic choice.

## 2. Experimental Section

### 2.1. Cohort of Patients

After obtaining informed consent, peripheral blood (PB) and bone marrow (BM) were collected at diagnosis and during follow-up from 11 CML patients: 3 patients with e13a3, 2 with e14a3, and 6 with e19a2. A total of 65 RNA samples (13 BM and 52 PB) were collected from three Italian diagnostic laboratories. The mean time from diagnosis was 26 months, ranging from 1 to 104 months of follow-up. Total RNA was reverse transcribed to complementary DNA using M-MLV reverse transcriptase, RNAse inhibitor, and random hexamer primers (respectively, #28025013, #N8080119, and #N8080127, Thermo Fisher Scientific, Waltham, MA, USA), following the manufacturer’s recommendations.

The study was approved by the ethics committee of San Luigi Gonzaga Hospital (approval number: 212/2015).

### 2.2. Standard Molecular Analysis

To assess the type of transcripts, *BCR–ABL1* was sequenced by the Sanger method, using the primers described in van Dongen et al. [10].

Monitoring of the patients’ follow-up was routinely performed in each laboratory with NESTED PCR, as described by van Dongen et al. [10].

### 2.3. ddPCR Molecular Analysis

Different primers and MGB-probes were designed, flanking the *BCR–ABL1* breakpoints (e13a3, a14a3, and e19a2), by using Primer Express 3.0 (Thermo Fisher Scientific, Waltham, MA, USA). Primer efficiency was calculated for each transcript with RT–PCR (CFX96, BioRad, Hercules, CA, USA) by using serial dilutions of cDNA from positive patients at diagnosis. The specificity of each amplicon was evaluated by analyzing the melting curve. Furthermore, we checked that selected primers and probes, listed in Table 1, do not amplify canonical *BCR–ABL1* transcripts (e13a2 and e14a2). The *ABL1* (#Hs01104728_m1, Thermo Fisher Scientific, Waltham, MA, USA) gene was used as an internal control.

The *BCR–ABL1* fusion and *ABL1* transcripts were tested in a multiplex, where probes for *BCR–ABL1* detection were labeled with FAM^TM^, while the probe for *ABL1* detection was labeled with VIC® fluorochrome in the same well. Each replicate was partitioned into ~20,000 droplets by a droplet generator (QX200™ Droplet Generator, BioRad, Hercules, CA, USA) and each droplet was amplified by using ddPCR™ Supermix for Probes (No dUTP; BioRad, Hercules, CA, USA) and the thermal cycling conditions suggested by the manufacturer. Final concentrations of custom primers and probes are listed in Table 1, while the *ABL1* assay was diluted following the manufacturer’s indications. Each sample was then loaded onto the QX200™ Droplet Reader (BioRad, Hercules, CA, USA), and ddPCR data were analyzed with QuantaSoft™ analysis software (version 1.7.4, BioRad, Hercules, CA, USA).

Each sample was analyzed in triplicate, and 100 ng of initial RNA were loaded for each well. The target concentration in each sample was expressed as a percentage of *BCR–ABL1/ABL1*. The limit of detection (LoD) of each assay was calculated mixing the RNA of samples without *BCR–ABL1* translocation with an increasing amount of RNA from patients with atypical transcripts (0%, 0.001%, 0.01%, 0.1%, 1%, and 10%).

## 3. Results

### 3.1. ddPCR Assays Assessment

**Figure 1:**
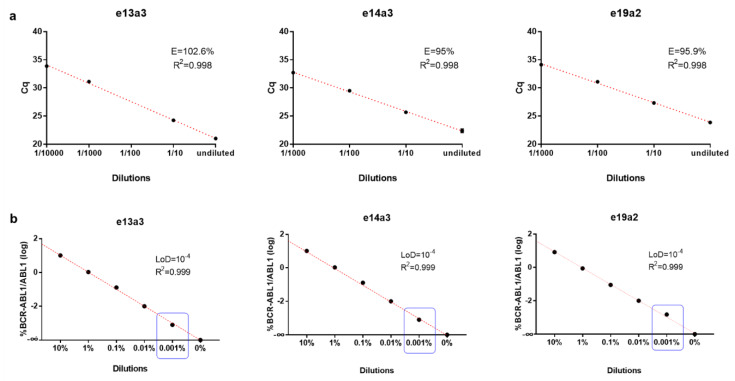
(**a**) Primers efficiency calculated by using RT–PCR. The ten-fold serial dilutions are linear over four/five logs. Data represent two replicates of each dilution. (E: efficiency; R^2^: coefficient of correlation). (**b**) Limit of detection (LoD) estimation of ddPCR assays assessed by mixing at different percentage positive patient’s RNA with a pool of RNA without BCR–ABL1 fusion transcript (10%, 1%, 0.1%, 0.01%, 0.001%, and 0%). LoD for each assay is highlighted in blue boxes.

Among the designed primers, we selected the best sets based on the evaluation of the efficiency. Selected primers showed high linearity (coefficient of correlation R^2^ = 0.9988, 0.9879 and 0.9984 for e13a3, e14a3 and e19a2, respectively) and efficiency (E = 102.6%, 95%, and 95.9% for e13a3, e14a3, and e19a2, respectively; Figure 1a). Successively, to estimate the LoD of the ddPCR assays, we simulated different MRD patients’ conditions. In detail, we mixed at different concentration (10%, 1%, 0.1%, 0.01%, 0.001%, and 0%) RNA from positive patients with a pool of RNA without *BCR–ABL1* fusion transcript. The results indicated that our assays could quantify a reduction of the *BCR–ABL1* transcript of up to 10^-4^. No positive droplets were detected in the sample used as a negative control (0% *BCR–ABL1* transcript). Furthermore, the curves obtained showed very high linearity (coefficient of correlation R^2^ = 0.999 for all the assays; Figure 1b). Our data indicated that ddPCR assays also allow the detection of low levels of *BCR–ABL1* transcript (0.001%), with high specificity.

### 3.2. BCR–ABL1 Evaluation in Patients

**Figure 2:**
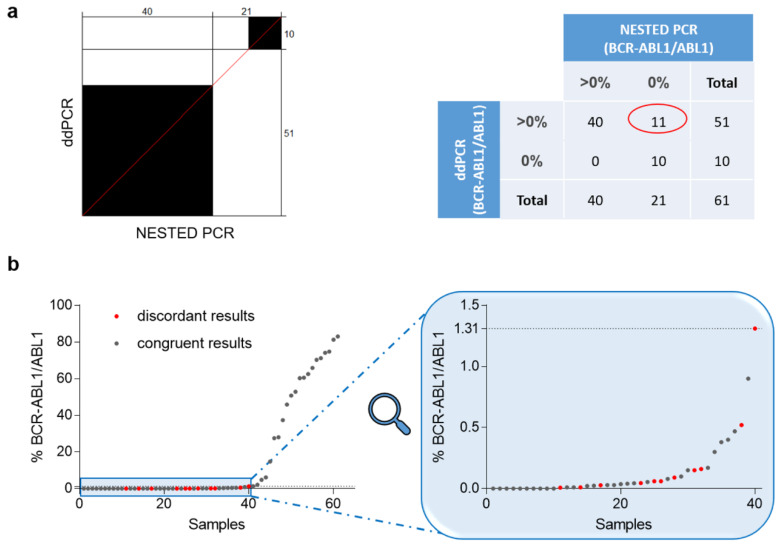
(**a**) Agreement chart for comparing *BCR–ABL1* level evaluation techniques. In agreement plots, the black blocks indicate the accordance of results between ddPCR and NESTED PCR. The 2 × 2 table alongside underlines the differences between ddPCR and NESTED PCR, reporting the number of samples positives (*BCR–ABL1/ABL1* >0%) and negatives (*BCR–ABL1/ABL1* equal to 0%) obtained with both the methods. (**b**) The graphs indicate all the percentages of *BCR–ABL1/ABL1* identified in CML patients with ddPCR and sorted from 0% to 83%. Zoom of the region under 1.32% of *BCR–ABL1/ABL1* is shown in the light blue box. Grey dots: congruent results; red dots: discordant results (samples positive in ddPCR, but negative in NESTED PCR).

The *BCR–ABL1* fusion transcript was evaluated in patients’ samples, at diagnosis, and during follow-up by NESTED PCR, performed routinely by each laboratory, and by ddPCR. ddPCR generated valuable data for all the samples analyzed; by contrast, NESTED PCR failed to evaluate 4 of these, probably due to the quality of RNA. ddPCR and NESTED PCR identified congruently 10 negative and 40 positive samples, while 11 were identified as positive only in ddPCR. To note, all these incongruently samples showed a low percentage of *BCR–ABL1/ABL1*, ranging between 0.008% and 1.31%. All the non-concordant samples were re-evaluated a second time with both the assays and all the results of the first analysis were confirmed. The agreement between ddPCR and NESTED PCR was evaluated by McNemar’s test that highlighted a marked difference in the proportion of disagreement data (*p* <0.01; Figure 2a). Moreover, the discriminatory ability of NESTED PCR was random in the ranges between 0.008% and 1.31% of the *BCR–ABL1/ABL1* transcript, suggesting reduced robustness of the NESTED PCR method near 1% of atypical transcripts (Figure 2b).

**Figure 3:**
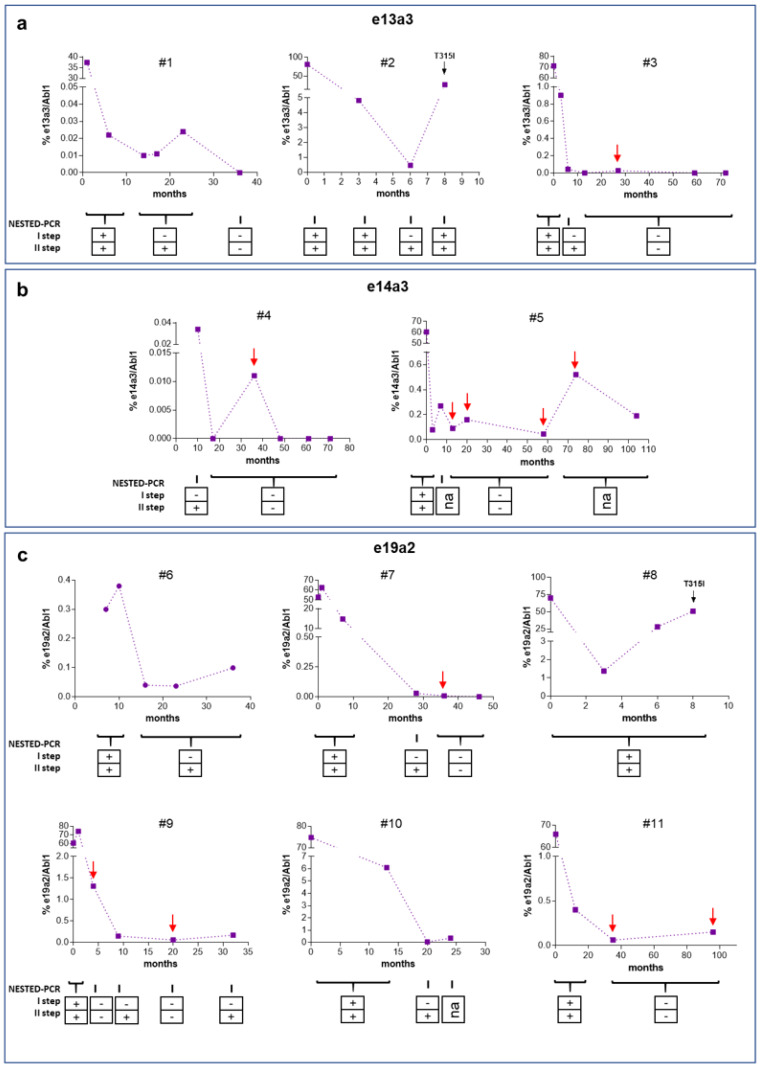
Results of ddPCR analysis for *BCR–ABL1* quantification of patients with e13a3, e14a3, and e19a2 fusion transcripts. The result of the NESTED PCR is indicated under each follow-up point. +: positive; −: negative; na: not available. Red arrows highlight discordant results between ddPCR and NESTED PCR. (**a**) e13a3 patients; (**b**) e14a3 patients; (**c**) e19a2 patients.

Results of the ddPCR analysis for *BCR–ABL1* quantification of patients with e13a3, e14a3, and e19a2 are shown in Figure 3. Seven points of follow-up have both BM and PB samples, and we found that results were concordant, confirming the data published in the literature [11,12]. For these cases, we only reported the PB results in the graphs for an easier interpretation. A summary of all the molecular analysis is reported in Supplementary Table S1.

Among the e13a3 group, Patient 1 showed a decreased expression of *BCR–ABL1* with a percentage of transcript less than 0.1% that was no longer detectable 36 months after TKI therapy; Patient 2 acquired the *T315I* mutation after 8 months of TKI therapy, while Patient 3 had a constant reduction in BCR–ABL1 levels, undetectable from months 59 to 72 (negative for more than 1 year) of TKI treatment (Figure 3a).

In the e14a3 group, Patient 4 showed an optimal response to TKI treatment, with a percentage of *BCR–ABL1* of 0.01% only at month 36 after therapy and was no more detectable from months 48 to 71 (negative for about 2 years). Patient 5 showed *BCR–ABL1* levels from 0.08% to 0.52% during all the time of TKI administration (104 months; Figure 3b).

Among the e19a2 group, only Patient 7 achieved a *BCR–ABL1/ABL1* level under 0.01% after 36 months and became negative after 46 months of TKI treatment (DMR for about 1 year); Patient 8 acquired *T315I* mutation; all the other patients showed a reduction in *BCR–ABL1* levels that remained detectable by ddPCR during the entire follow-up, while NESTED PCR failed to detect several of these cases (Figure 3c).

The data indicated that our ddPCR assays were more effective than NESTED PCR in defining the molecular response in these patients, managing to identify three patients who have achieved and maintained a DMR for at least one year.

## 4. Discussion

The introduction of TKI has completely changed CML management, helping to improve the response rate, survival, and quality of life of patients [13]. *BCR–ABL1* levels represent the molecular marker for the evaluation of MRD, defining the depth of molecular remission, and guiding clinical decisions [14]. Differently from the majority of CML patients, 1–2% of these show breakpoints on chromosomes 9 and 22 located in unusual regions, giving rise to atypical transcripts.

We describe a new method based on ddPCR for the absolute quantification of *BCR–ABL1* levels in CML patients with atypical transcripts. Despite the fact that NESTED PCR and Sanger sequencing remain useful to diagnose and identify the *BCR–ABL1* transcripts, we demonstrate that our ddPCR assays are effective in defining the molecular response in these patients, improving clinical information and guiding therapeutic decisions. Our assay shows good efficiency and a very low LoD, allowing us to also identify positive samples when the *BCR–ABL1* level was so low that NESTED PCR was inaccurate. In particular, NESTED PCR was imprecise when the transcript level was lower than 1%, and the positivity could only be detected with a second PCR step, suggesting that it is a not suitable technique for a precise MRD assessment. By contrast, ddPCR results can be considered more reliable because positive samples showed a percentage of *BCR–ABL1/ABL1* greater than LoD identified in the setting analysis.

Recent studies have shown that discontinuing TKI treatment in patients who have achieved a stable DMR could decrease the cost of therapy and altogether reduce the treatment side effects, increasing the quality of life of CML patients [4].

Despite the fact that current guidelines do not recommend discontinuation for patients with atypical transcripts, our group has previously described 7 cases that have successfully discontinued the treatment due to severe comorbidities [6], also suggesting the need for quantitative methodologies for the atypical transcripts. Of note, we identified at least 3 patients (subject 3 e13a3, 4 e14a3 and 7 e19a2) who achieved and maintained a DMR for at least one year. These patients could represent good candidates for MRD monitoring in view of a possible suspension of TKI treatment.

In conclusion, we have described a new highly sensitive and specific method for molecular monitoring of atypical *BCR–ABL1* fusion transcripts in CML patients that helps in the definition of MRD levels to better delineate a therapeutic strategy and the optimal time for discontinuation.

## Figures and Tables

**Table 1:** Primers and probes used for droplet digital PCR (ddPCR) assays.

Primers/Probes	Sequences 5′-3′	Final PCR Concentrations (nM)
e13 forward	TCGTGTGTGAAACTCCAGACTGT	900
e14 forward	CCACTGGATTTAAGCAGAGTTCAA	900
a3 reverse	CTTCACACCATTCCCCATTG	900
a3 probe (FAM™-MGB)	TGAAAAGCTCCGGGTCT	250
e19 forward	CACTGAAGGCAGCCTTCGA	900
a2 reverse	GAGGCTCAAAGTCAGATGCTACTG	900
a2 probe (FAM™-MGB)	TCAAAGCCCTTCAGCG	250

## Supplementary Materials

The following are available online at https://www.mdpi.com/2077-0383/9/5/1457/s1. Table S1: Summary of all molecular analyses.
