# Supplementary material for: Novel Multiplex Droplet Digital PCR Assays to Monitor Minimal Residual Disease in Chronic Myeloid Leukemia Patients Showing Atypical BCR-ABL1 Transcripts
Source: J Clin Med. 2020 May 13;9(5):1457. doi: 10.3390/jcm9051457 (PMC7290999; doi:10.3390/jcm9051457)
Supplement: Supplementary file 1 [file jcm-09-01457-s001.pdf]

Supplementary Table S1. Summary of all molecular analyses.

BCR-ABL1 transcript	Patient ID	TFD (m)	PB/BM	NESTED PCR	ddPCR (%*BCR-ABL1*/*ABL1*)
e13a3	#1	1	BM	I step	37.4
e13a3	#1	6	PB	I step	0.022
e13a3	#1	14	PB	II step	0.01
e13a3	#1	17	PB	II step	0.011
e13a3	#1	23	PB	II step	0.024
e13a3	#1	36	PB	II step	0
e13a3	#2	0	BM	I step	81.3
e13a3	#2	3	PB	I step	4.83
e13a3	#2	6	PB	II step	0.47
e13a3	#2	8	PB	I step	27.5
e13a3	#3	0	PB	I step	71.2
e13a3	#3	3	PB	I step	0.9
e13a3	#3	6	BM	II step	0.044
e13a3	#3	13	PB	negative	0
e13a3	#3	27	PB	negative	0.028
e13a3	#3	59	PB	negative	0
e13a3	#3	72	PB	negative	0
e14a3	#4	10	PB	negative	0
e14a3	#4	10	BM	II step	0.034
e14a3	#4	17	PB	negative	0
e14a3	#4	36	PB	negative	0.011
e14a3	#4	48	PB	negative	0
e14a3	#4	61	PB	negative	0
e14a3	#4	71	PB	negative	0
e14a3	#5	0	BM	I step	60.3
e14a3	#5	3	PB	I step	0.079
e14a3	#5	7	PB	na	0.27
e14a3	#5	13	PB	negative	0.09
e14a3	#5	20	PB	negative	0.16
e14a3	#5	58	PB	negative	0.045
e14a3	#5	74	PB	negative	0.52
e14a3	#5	104	BM	na	0.19
e14a3	#5	104	PB	na	0.12
e19a2	#6	7	PB	I step	0.3
e19a2	#6	10	PB	I step	0.38
e19a2	#6	16	PB	II step	0.04
e19a2	#6	23	PB	II step	0.037
e19a2	#6	36	PB	II step	0.099
e19a2	#7	0	PB	I step	52.9
e19a2	#7	1	BM	I step	62.5
e19a2	#7	7	PB	I step	14.8
e19a2	#7	28	PB	II step	0.029
e19a2	#7	36	PB	negative	0.008
e19a2	#7	46	PB	negative	0
e19a2	#8	0	PB	I step	70.3
e19a2	#8	0	BM	I step	83
e19a2	#8	3	PB	I step	1.37

e19a2	#8	3	BM	I step	2.12
e19a2	#8	6	PB	I step	28
e19a2	#8	6	BM	I step	45.9
e19a2	#8	8	PB	I step	50.8
e19a2	#9	0	PB	I step	60.5
e19a2	#9	0	PB	I step	74.1
e19a2	#9	4	PB	negative	1.31
e19a2	#9	9	PB	II step	0.15
e19a2	#9	20	PB	negative	0.06
e19a2	#9	32	PB	II step	0.17
e19a2	#10	0	BM	I step	74.8
e19a2	#10	13	PB	I step	6.1
e19a2	#10	20	PB	II step	0.054
e19a2	#10	24	PB	na	0.36
e19a2	#11	0	BM	I step	65.9
e19a2	#11	12	BM	I step	0.4
e19a2	#11	35	PB	negative	0.06
e19a2	#11	96	PB	negative	0.15

I step, sample positive at I PCR step; II step, sample positive at II PCR step; negative; sample negative at both PCR steps; red rows highlight discordant results. Abbreviation: TFD (m), time from diagnosis (months); na, not available; ddPCR, droplet digital PCR.
